# The Study to Investigate COVID-19 Infection in People Living in Ireland (SCOPI): A seroprevalence study, June to July 2020

**DOI:** 10.2807/1560-7917.ES.2021.26.48.2001741

**Published:** 2021-12-02

**Authors:** Laura Heavey, Patricia Garvey, Aoife M Colgan, Lelia Thornton, Jeff Connell, Thomas Roux, Meadhbh Hunt, Fiona O'Callaghan, Fiona Culkin, Mary Keogan, Nuala O'Connor, Margaret B O'Sullivan, Siobhán O’Sullivan, Michele Tait, Cillian F De Gascun, Derval Igoe

**Affiliations:** 1 Health Protection Surveillance Centre, Health Service Executive, Dublin, Ireland; 2 National Virus Reference Laboratory, University College Dublin, Dublin, Ireland; 3 Methodology Division, Central Statistics Office, Cork, Ireland; 4 Department of Clinical Immunology, Beaumont Hospital and Royal College of Surgeons in Ireland, Dublin, Ireland; 5 Irish College of General Practitioners, Dublin, Ireland; 6 Department of Public Health, Health Service Executive South, Cork, Ireland; 7 Office of the Chief Medical Officer, Department of Health, Dublin, Ireland; 8 Office of the Chief Operations Officer, Health Service Executive, Dublin, Ireland

**Keywords:** Cross-sectional studies, Seroepidemiologic studies, COVID-19, SARS-CoV-2

## Abstract

**Background:**

Robust data on SARS-CoV-2 population seroprevalence supplement surveillance data in providing evidence for public health action.

**Aim:**

To conduct a SARS-CoV-2 population-based seroprevalence survey in Ireland.

**Methods:**

Using a cross-sectional study design, we selected population samples from individuals aged 12–69 years in counties Dublin and Sligo using the Health Service Executive Primary Care Reimbursement Service database as a sampling frame. Samples were selected with probability proportional to the general population age–sex distribution, and by simple random sampling within age–sex strata. Antibodies to SARS-CoV-2 were detected using the Abbott Architect SARS-CoV-2 IgG Assay and confirmed using the Wantai Assay. We estimated the population SARS-CoV-2 seroprevalence weighted for age, sex and geographic area.

**Results:**

Participation rates were 30% (913/3,043) and 44% (820/1,863) in Dublin and Sligo. Thirty-three specimens had detectable SARS-CoV-2 antibodies (1.9%). We estimated weighted seroprevalences of 3.12% (95% confidence interval (CI): 2.05–4.53) and 0.58% (95% CI: 0.18–1.38) for Dublin and Sligo, and 1.69% (95% CI: 1.13–2.41) nationally. This equates to an estimated 59,482 (95% CI: 39,772–85,176) people aged 12–69 years nationally having had infection with SARS-CoV-2, 3.0 (95% CI: 2.0–4.3) times higher than confirmed notifications. Ten participants reported a previous laboratory-confirmed SARS-CoV-2 -infection; eight of these were antibody-positive. Twenty-five antibody-positive participants had not reported previous laboratory-confirmed infection.

**Conclusion:**

The majority of people in Ireland are unlikely to have been infected with SARS-CoV-2 by June–July 2020. Non-pharmaceutical public health measures remained key pending widespread availability of vaccination, and effective treatments.

## Introduction

Case-based surveillance may considerably underestimate the level of severe acute respiratory syndrome coronavirus 2 (SARS-CoV-2) infection, as mild or asymptomatic cases may not seek healthcare and testing. Serological detection of SARS-CoV-2-specific antibodies can better estimate the true number of cases [1].

In spring 2020, the Irish National Public Health Emergency Team (NPHET), which advises the Irish Government, requested that a population-based seroprevalence survey be conducted with the aim of informing the public health response. Considerations when embarking on seroprevalence studies, especially for a recently emerging virus like SARS-CoV-2, include the timing of the study, since early testing may result in false negative results before seroconversion, but late testing may also miss cases if there is rapid antibody decline. A wide-ranging evidence summary indicated that IgG antibodies are typically produced in the 2 weeks following an infection with SARS-CoV-2 and persist for at least 3 months [2]. Other studies showed antibody persistence for at least 4 months [3,4]. Also, individuals vary in their antibody response to SARS-CoV-2, both in the level of antibody produced and to which viral protein target (nucleoprotein or spike protein) their antibodies are predominantly directed. It is possible that individuals who had milder disease or were asymptomatic are less likely to develop a detectable antibody response in serum [2]. Another consideration is the assay target and assay performance, with data suggesting that assays targeting the spike glycoprotein may be preferable in people with low level antibody responses [5].

This study, entitled ‘The Study to Investigate COVID-19 (coronavirus disease) Infection in People Living in Ireland’ or SCOPI, was conducted in June–July 2020, after the first wave of COVID-19 and before any appreciable decline in antibody levels should have occurred. Antibodies were measured using two validated commercial assays, the first targeting the nucleoprotein and a second targeting the spike protein of SARS-CoV-2, the latter of which was used for specimens exceeding or within 25% of the cut-off value in the first assay.

The primary objective of the study was to measure the prevalence of antibodies to SARS-CoV-2 in a representative sample of the population in two geographically defined regions (selected to represent counties with high and low reported cumulative incidence of PCR-confirmed SARS-CoV-2 infection, respectively), in order to estimate the national prevalence of SARS-CoV-2 infection. Our secondary objective was to examine the relationship between the presence of SARS-CoV-2 antibodies and the self-reporting of symptoms or previous diagnosis of COVID-19.

## Methods

### Study design and population

Using a cross-sectional study design, based on the World Health Organization (WHO) protocol ‘Population-based age-stratified sero-epidemiological investigation protocol for COVID-19 virus infection’ [6], we selected random samples of people in each of two defined geographic areas, counties Dublin (population aged 12–69 years: n = 1,021,801) and Sligo (population aged 12–69 years: n = 47,718), which have high and low incidence of notified COVID-19 cases, respectively, which together represent 30% of the national population in this age group. Selection of these two counties was non-random, and took into account the practicalities of the setting up the study. We had no reason to believe there was a bias in reported detection of COVID-19 between the two counties as there were nationally agreed clinical criteria for testing for COVID, community testing hubs had been established across the country and testing was free. We excluded those under 12 years as phlebotomy is difficult in young children, and people aged 70 and older as they had been advised to stay at home during this study period.

There is no national population register in Ireland available for research purposes; therefore, we used the Health Service Executive (HSE) Primary Care Reimbursement Service (PCRS) database (https://www.hse.ie/eng/staff/pcrs/about-pcrs) as a sampling frame. The PCRS database covers ca 60% of the population in Ireland, and includes those who avail of primary care payment schemes. We selected a sample proportional to the size of the age group and sex strata in the general population for each county [7]. Within age–sex strata, we selected participants using simple random sampling. We estimated sample sizes assuming a seroprevalence of 6% and a precision of 1.2% for Dublin (n = 1,600) and a seroprevalence of 1.5% and a precision of 0.75% for Sligo (n = 1,000), and doubled the numbers invited to take account of anticipated non-response and ineligibility.

### Recruitment

Recruitment took place in the week of 15 June 2020. The invitation letter in English and Irish was accompanied by a detailed information leaflet. Information was also available on a study website, www.hse.ie/scopi. Invitees were asked to respond by phone, text message, or email. We sent one reminder letter to non-responders 2 weeks after the initial letter. The invitation letter was available on the study website in six additional languages. A translation service, sign language interpreters, and video calls for lip reading were available as required.

### Procedure

We administered a short questionnaire (https://www.hpsc.ie/a-z/respiratory/coronavirus/novelcoronavirus/scopi/SCOPI%20Questionnaire.pdf ) by phone to those who agreed to take part. This included questions about demographic characteristics, previous diagnosis of COVID-19, close contact with a COVID-19 case, and symptoms suggestive of COVID-19 experienced since the end of February 2020. Of note, only five symptoms (fever, cough, shortness of breath, loss sense of smell or taste) were asked about from the national COVID-19 case definition which at the time included six symptoms; dysgeusia was not included in the SCOPI questionnaire. We made appointments for participants to attend an HSE clinic for blood sampling. We excluded participants from blood sampling if they were restricting their movements at the time of the study on medical advice, if they were a close contact of a COVID-19 case, or if they were ill with suspected or confirmed COVID-19 (Table 1) at the time of the blood test appointment.

**Table 1:** COVID-19 interim case definition in place for notification of COVID-19 cases at the time of the study, Ireland, June 2020

Classification criteria		Definition
Clinical criteria^a^		A patient with acute respiratory infection with sudden onset of at least one of the following: cough, fever^b^, shortness of breath OR sudden onset of anosmia^c^, ageusia^d^ and dysgeusia^e^ OR with severe acute respiratory infection (SARI) with fever and at least one sign/symptom of respiratory disease, e.g. cough, fever, shortness of breath AND requiring hospitalisation AND with no other aetiology that fully explains the clinical presentation
Epidemiological criteria		A patient with at least one of the following two epidemiological links: close contact^f^ with a confirmed COVID-19 case in the 14 days prior to onset of symptoms OR having been a resident or a staff member in the 14 days prior to onset of symptoms, in a residential institution for vulnerable people where ongoing COVID-19 transmission has been confirmed
Diagnostic imaging criteria		Radiological evidence showing lesions compatible with COVID-19
Laboratory criteria		Detection of SARS-CoV-2 nucleic acid in a clinical specimen
Case classification	Possible case	Any person meeting the clinical criteria
	Probable case	Any person meeting the clinical criteria with an epidemiological link OR meeting the diagnostic imaging criteria
	Confirmed case	Any person meeting the laboratory criteria

COVID-19: coronavirus disease; SARI: severe acute respiratory infection; SARS-CoV-2: severe acute respiratory syndrome coronavirus 2.

^a^ Clinical judgement should be applied in application of these criteria to determine who requires testing.

^b^ Fever may be subjective or confirmed by a healthcare worker (≥ 38^◦^C).

^c^ Loss of sense of smell.

^d^ Loss of sense of taste.

^e^ Distortion of sense of taste.

^f^ Close contact is defined as face-to-face contact at < 2 metres for longer than 15 minutes.

We collected all blood samples between 22 June 2020 and 16 July 2020. Participants were provided with their results by letter at the end of the study.

###  Laboratory analysis

At the phlebotomy clinic, whole blood samples were stored in EDTA blood sample tubes at 4°C and transferred by courier to the National Virus Reference Laboratory (NVRL) at University College Dublin (UCD) for SARS-CoV-2 antibody testing. The extracted serum was tested once, without any freeze-thaw cycles, in the Abbott Architect SARS-CoV-2 IgG Assay (Abbott Diagnostics, Chicago, United States (US)), which detects IgG targeting the nucleoprotein. The assay was performed in accordance with the manufacturer’s protocol. Sensitivity and specificity were 93.9% and 100%, respectively, according to a Public Health England evaluation [8].

Any specimen exceeding or within 25% of the cut-off value in the Abbott Assay was re-tested using the Wantai SARS-CoV-2 Ab ELISA Assay (Beijing Wantai Biological Pharmacy, Beijing, China), which detects total (IgG/IgM/IgA) antibody directed against the receptor-binding domain of the spike protein of SARS-CoV-2. The assay was performed in accordance with the manufacturer’s criteria. The sensitivity and specificity of the Wantai ELISA were both found to be 99% when evaluated [9].

Any samples testing positive using the Wantai assay were reported as SARS-CoV-2 antibody-positive; any samples testing negative using the Wantai assay were reported as SARS-CoV-2 antibody-negative. No external SARS-CoV-2 independent quality control was used during the laboratory investigation.

### Statistical analysis

We calculated the prevalence of SARS-CoV-2 antibodies and 95% confidence intervals (CI), overall and by age and sex for each county, weighted to adjust for varying response rates in age–sex strata, and using exact confidence intervals (Clopper–Pearson).

On 27 June 2020, the cumulative incidence rate for confirmed COVID-19 notifications in Dublin was 898 cases/100,000 population and in Sligo was 210 cases/100,000 population. Using a cut-off of the national cumulative incidence rate for COVID-19 notifications on 27 June (551 cases/100,000 population), we categorised all 26 counties in Ireland into ‘high’ and ‘low’ incidence counties. Seventeen counties including Sligo had cumulative incidence rates below the cut-off (median 291 cases/100,000 population (range: 151–497)). Nine counties including Dublin had cumulative incidence rates above the cut-off (median 811 cases/100,000 population (range: 610–1,241)).

We estimated the national prevalence of SARS-CoV-2 antibodies and 95% confidence intervals (CI), overall and by age and sex, weighted for the age–sex distribution of the population aged 12–69 years in the two groups of counties, by applying the seroprevalence obtained for the Dublin sample to produce weighted estimates for the population in all ‘high’ incidence counties and the seroprevalence obtained for the Sligo sample to produce weighted estimates for the population in all ‘low’ incidence counties.

We used the seroprevalence in the Dublin and Sligo samples and the estimated national seroprevalence to estimate the number of seropositive individuals in the population aged 12–69 years in Dublin, in Sligo, and in Ireland, respectively.

We compared the reported clinical history and COVID-19-like symptoms of seropositive and seronegative participants in the study population using Fisher’s Exact Test (unweighted analyses). We calculated the prevalence of SARS-CoV-2 antibodies in each county by symptom history, weighted for age and sex.

We used Stata SE-64 v15.1 (Stata Corporation, College Station, Texas, US), SAS Enterprise Guide version 7 statistical software (SAS Institute, Cary, North Carolina, US), and QGIS Desktop 3.12 for mapping (https://qgis.org).

### Ethical statement

The National COVID-19 Research Ethics Committee approved the study (ID: 20-NREC-COV-047). We obtained verbal consent during telephone interviews and written consent when the participant presented at the testing site. For participants under the age of 18 years, we obtained the consent of the parent or guardian and the assent of the child. We provided participants with the option of accessing decision-making assistance through an independent advocacy service.

## Results

### Response rates

**Figure 1:**
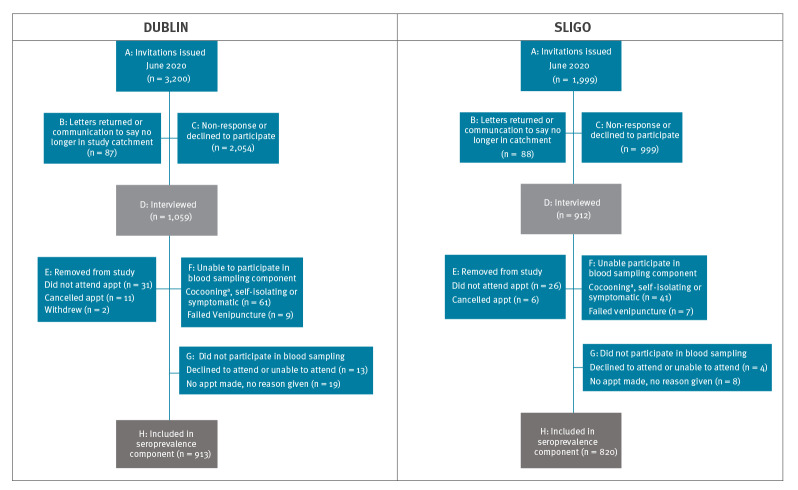
Participation and non-participation among SCOPI study invitees, Dublin and Sligo, Ireland, 22 June–16 July 2020 (n = 5,199)

Of 3,200 individuals invited to participate from Dublin, 3,113 were eligible for one or both components (questionnaire and blood test for seroprevalence) of the study (Figure 1). Of these, 1,059 completed a questionnaire, but 44 withdrew subsequently, giving a response rate of 33% (1,015/3,113). The participation rate in both components was 30% (913/3,043) (Figure 1 and Table 2).

Appt: appointment; COVID-19: coronavirus disease; SARS-CoV-2: severe acute respiratory syndrome coronavirus 2; SCOPI: The Study to Investigate COVID-19 (coronavirus disease) Infection in People Living in Ireland.

^a ^The term ‘cocooning’ was used to describe the request by government to individuals at higher risk of consequences from SARS-CoV-2 infection to stay at home most of the time. This included, e.g. older people (˃ 70 years) in the general population, people who had cancers and rare diseases, organ transplantation, chronic lung illnesses, and those who live in long-term care homes, among others.

The response rate for the questionnaire component of the study was calculated as (F + G + H)/(A − B). The response rate for the seroprevalence component of the study was calculated as H/(A − B − F).

**Table 2:** Demographic characteristics of the SCOPI sampling frame, invited study sample and study participants, Dublin and Sligo, Ireland, 22 June–16 July 2020 (n = 407,009)

Characteristics			PCRS sampling frame (n = 407,009)		Invited study sample^a^ (n = 5,199)		Participants in seroprevalence component of study^b^ (n = 1,733)	
County	Sex	Age (years)	n	%	n	%	n	%
Dublin	Male	12–19	32,956	9	200	6	42	5
		20–29	25,610	7	313	10	67	7
		30–39	28,318	7	374	12	78	9
		40–49	36,499	10	289	9	81	9
		50–59	31,148	8	225	7	69	8
		60–69	23,981	6	169	5	84	9
	Female	12–19	31,263	8	193	6	48	5
		20–29	31,942	8	325	10	80	9
		30–39	39,463	10	389	12	93	10
		40–49	41,835	11	296	9	108	12
		50–59	33,192	9	241	8	84	9
		60–69	27,613	7	186	6	79	9
Dublin total		12–69	383,820	100	3,200	100	913	100
Sligo	Male	12–19	1,912	8	150	8	50	6
		20–29	1,801	8	140	7	37	5
		30–39	1,685	7	181	9	51	6
		40–49	2,140	9	184	9	71	9
		50–59	2,003	9	177	9	71	9
		60–69	1,718	7	154	8	75	9
	Female	12–19	1,826	8	143	7	58	7
		20–29	1,852	8	145	7	56	7
		30–39	2,063	9	197	10	88	11
		40–49	2,245	10	187	9	97	12
		50–59	1,989	9	185	9	93	11
		60–69	1,955	8	156	8	73	9
Sligo total		12–69	23,189	100	1,999	100	820	100

PCRS: Health Service Executive Primary Care Reimbursement Service. SCOPI: The Study to Investigate COVID-19 (coronavirus disease) Infection in People Living in Ireland.

^a^ Reflected county population by age and sex.

^b^ Of the invited participants who completed the SCOPI questionnaire, this subset also provided a blood specimen.

Of 1,999 individuals invited to participate from Sligo, 1,911 were eligible for either or both components of the study (Figure 1). Of these, 912 completed a questionnaire, but 32 withdrew subsequently, giving a response rate of 46% (880/1,911). The participation rate for both components was 44% (820/1,863) (Figure 1 and Table 2).

By design, the invited sample reflected the county population by age and sex. In addition to the response rate differing by county, the response rate also differed by age and sex: 27% (748/2,750) of invitees aged 12–39 years participated in both study components compared with 40% (985/2,449) of invitees aged 40–69 years; and 30% (776/2,556) of male invitees participated compared with 36% of females (957/2,643) (Table 2).

### Laboratory findings

Of the 1,733 participants who provided blood samples, 33 specimens were deemed to have detectable antibodies to SARS-CoV-2 (1.9%). Thirty-six specimens exceeded the Abbott test assay threshold. Five of these were subsequently deemed negative following a Wantai test, while an additional two specimens that were borderline when first tested using the Abbott test were deemed positive using the Wantai test.

### Seroprevalence in Dublin and Sligo

In Dublin, there were 28 specimens confirmed as seropositive, corresponding to a weighted seroprevalence of 3.12% (95% CI: 2.05–4.53) (Table 3). Based on these findings, we estimated that 31,880 (95% CI: 20,947–46,288) people in the population aged 12–69 years in Dublin had an infection with SARS-CoV-2 at that time, 3.4 times (95% CI: 2.3–5.0) higher than the number of laboratory-confirmed COVID-19 cases aged 12–69 years in Dublin notified by the end of the study (16 July). Seroprevalence was not significantly different by age group or by sex.

**Table 3:** Estimated SARS-CoV-2 seroprevalence by age and sex in the population aged 12–69 years, Dublin and Sligo, Ireland, 22 June–16 July 2020 (n = 33)

Category		Ireland			Dublin			Sligo		
		Number seropositive (n)	Weighted prevalence (%)	95% CI	Number seropositive (n)	Weighted prevalence (%)	95% CI	Number seropositive (n)	Weighted prevalence (%)	95% CI
Sex	Females	20	1.84	1.08–2.90	18	3.61	2.12–5.71	2	0.44	0.05–1.59
	Males	13	1.53	0.79–2.67	10	2.61	1.23–4.82	3	0.72	0.12–2.27
Age group (years)	12–19	3	1.44	0.28–4.25	2	2.23	0.26–7.92	1^a^	0.26	0.004–1.56
	20–29	7	2.32	0.81–5.12	7	4.65	1.86–9.42			
	30–39	5	1.42	0.42–3.46	5	2.98	0.97–6.84			
	40–49	7	1.75	0.66–3.71	6	3.09	1.12–6.67	4^b^	0.88	0.24–2.25
	50–59	5	1.53	0.49–3.58	3	1.93	0.39–5.58			
	60–69	6	1.69	0.57–3.83	5	3.22	1.05–7.34			
Population total	12–69	33	1.69	1.13–2.41	28	3.12	2.05–4.53	5	0.58	0.18–1.38

CI: confidence interval; SARS-CoV-2: severe acute respiratory syndrome coronavirus 2.

^a^ One age group of 12–39 years was analysed because of low numbers of positive samples.

^b^ One age group of 40–69 years was analysed because of low numbers of positive samples.

In Sligo, there were five specimens confirmed as seropositive, corresponding to a weighted seroprevalence of 0.58% (95% CI: 0.18–1.38) (Table 3). Based on these findings, we estimate that 277 (95% CI: 86–659) people in the population aged 12–69 years in Sligo had an infection with SARS-CoV-2 by that time, 2.4 times (95% CI: 0.8–5.8) higher than the number of laboratory-confirmed COVID-19 cases aged 12–69 years notified in Sligo by 16 July. Seroprevalence was not significantly different by age group or by sex.

### National seroprevalence estimate

**Figure 2:**
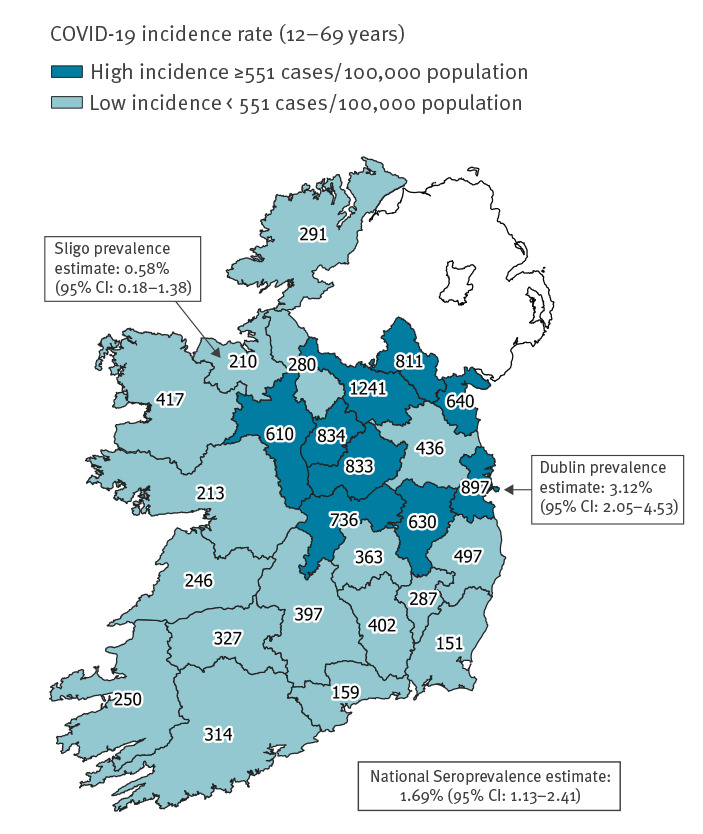
National COVID-19 incidence rates by county and SARS-CoV-2 seroprevalence estimates, Sligo and Dublin, Ireland, 22 June–16 July 2020

COVID-19: coronavirus disease; SARS-CoV-2: severe acute respiratory syndrome coronavirus 2.

The crude incidence rates of notified confirmed COVID-19 cases, and high/low incidence categories for all counties, are illustrated in Figure 2. Based on these categories, the results of the Dublin and Sligo serosurveys, and the age–sex distribution of the county populations, we estimated a weighted national seroprevalence of 1.69% (95% CI: 1.13–2.41) (Table 3). This would translate to an estimated 59,482 (95% CI: 39,772–85,176) people in the national population aged 12–69 years who had been infected with SARS-CoV-2 by that time, 3.0 times (95% CI: 2.0–4.3) higher than the number of confirmed COVID-19 cases aged 12–69 years notified in Ireland by 16 July. The national seroprevalence estimates were not significantly different by age or by sex.

Date source for notification data: Computerised Infectious Disease Reporting (CIDR) system.

### Infection fatality ratio

There were 190 deaths among 19,652 COVID-19 cases aged 12–69 years notified up to 16 July, which equates to a case fatality ratio of 0.97%. If, as this study suggests, there were an estimated 59,482 cases nationally in the time period in this age group, this would suggest a revised estimated infection fatality ratio of 0.32% (95% CI: 0.22–0.48).

### Seropositivity and previous diagnosis

Ten of the 1,733 SARS-CoV-2-seropositive participants reported that they had a laboratory-confirmed SARS-CoV-2 infection in the previous 4 months, eight of whom had detectable anti-SARS-CoV-2 antibodies in this study (Table 4). Two of the participants reporting a laboratory-confirmed SARS-CoV-2 infection had no detectable anti-SARS-CoV-2 antibodies; neither had been hospitalised with COVID-19. These two participants were diagnosed in March and early May 2020, respectively.

**Table 4:** Number and proportion of respondents with COVID-19 symptoms by SARS-CoV-2 seropositivity status, Dublin and Sligo, Ireland, 22 June–16 July 2020 (n = 1,732)

Symptom/condition	Seropositive respondents (n = 33)			Seronegative respondents^a^ (n = 1,699)			p value^b^
	Yes	No	Not specified	Yes	No	Not specified	
Previous laboratory-confirmed SARS-CoV-2 infection	8	25	0	2	1,678	19	< 0.001
Close contact with someone with suspected or confirmed SARS-CoV-2 infection since February 2020	13	16	4	133	1,460	106	< 0.001
Fever	12	21	0	102	1,584	13	< 0.001
Chills	8	25	0	109	1,580	10	0.001
Fatigue	17	16	0	301	1,376	22	< 0.001
Muscle aches	14	19	0	186	1,501	12	< 0.001
Sore throat	6	27	0	261	1,427	11	0.629
Cough	13	20	0	293	1,401	5	0.004
Runny nose	7	25	1	304	1,385	10	0.641
Shortness of breath	6	26	1	164	1,527	8	0.123
Chest pain	5	27	1	97	1,598	4	0.037
Other respiratory symptoms	1	31	1	71	1,619	9	1.000
Headache	15	18	0	319	1,365	15	0.001
Loss of sense of smell (anosmia)	9	23	1	47	1,639	13	< 0.001
Loss of sense of taste (ageusia)	10	23	0	48	1,644	7	< 0.001
Loss of either sense of smell or taste (composite of previous two variables)	11	22	0	67	1,617	15	< 0.001
Sought medical attention for symptoms	18	12	0	193	1,432	74	< 0.001
Admitted to hospital for symptoms^c^	2	28	0	15	1,594	90	0.037
Consistent with case definition^d^	24	9	0	434	1,265	0	< 0.001
Asymptomatic	3	30	0	890	809	0	< 0.001

COVID-19: coronavirus disease; SARS-CoV-2: severe acute respiratory syndrome coronavirus 2.

^a^ One serosurvey respondent was omitted because no information on symptoms existed.

^b^ Fishers exact test unweighted analyses

^c^ Only the symptomatic seropositive respondents were included for the seropositive respondents (n = 30).

^d^ Reported at least one of the following: fever, cough, shortness of breath, loss sense of smell or taste.

Twenty-five of the 33 participants with detectable anti-SARS-CoV-2 antibodies did not report a previous laboratory-diagnosed SARS-CoV-2 infection.

### Seropositivity and reported symptoms

Of the 33 participants with antibodies to SARS-CoV-2, 24 reported at least one of five COVID-19 associated symptoms listed within the national COVID-19 case definition of the time (fever, cough, shortness of breath, loss sense of smell or taste). Of note, the national COVID-19 case definition at the time of the study included six symptoms: dysgeusia was not included in the SCOPI questionnaire. This was significantly higher than the proportion of seronegative participants 434/1,699 who reported symptoms consistent with the COVID-19 case definition (p < 0.001) (Table 4). Of the 24 seropositive participants who reported symptoms, six were previously diagnosed with a SARS-CoV-2 infection and a further five reported previous contact with a suspected or confirmed COVID-19 case.

Six seropositive participants, defined in this study as paucisymptomatic, reported other symptoms associated with COVID-19, such as fatigue and muscle aches, but did not report symptoms consistent with the case definition. A significantly lower proportion of seropositive participants (3/33) reported having no symptoms of COVID-19 compared with 890/1,699 of seronegative participants (p < 0.001) (Table 4).

Four of the symptoms listed in the COVID-19 case definition (Table 1) (and included in the SCOPI questionnaire) were significantly associated with seropositivity when assessed alone; specifically, 11/33 of seropositive participants reported loss of either sense of smell (anosmia) or taste (ageusia), compared with only 67/1,684 seronegative participants (p < 0.001) (Table 4). Shortness of breath was not significantly associated with the detection of anti-SARS-CoV-2 when all participants were included in the analyses (p = 0.123); however, a significant association with anti-SARS-CoV-2 detection was observed when shortness of breath analyses were confined to participants aged 40–69 years (p = 0.014; 5/17 vs 84/962).

Using the Dublin participant dataset, the weighted seroprevalence was lowest among those who reported no symptoms (0.55%; 95% CI: 0.06–2.01), higher among the paucisymptomatic group (2.7%; 95% CI: 0.86–6.31), and highest among those who reported symptoms consistent with the COVID-19 case definition (7.4%; 95% CI: 4.5–11) (Table 5). This trend in increasing seropositivity with increasing degree of symptomatology remained when younger (12–39 years), older (40–69 years), and male and female participants were examined separately in the Dublin dataset (data not shown). A similar finding was obtained using data from the Sligo participants; however, the CIs between all levels overlapped indicating the finding was not statistically significant, likely because of the lower overall seroprevalence in this group.

**Table 5:** Estimated SARS-CoV-2 seroprevalence, by reported experience of COVID-19 symptoms, Dublin and Sligo, Ireland, 22 June–16 July 2020 (n = 33)

COVID-19 symptom level	Dublin			Sligo		
	Seropositive (n = 28)	Weighted prevalence (%)	95% CI	Seropositive (n = 5)	Weighted prevalence (%)	95% CI
Asymptomatic	2	0.55	0.06–2.01	1	0.19	0.003–1.16
Paucisymptomatic^a^	5	2.71	0.86–6.31	1	0.57	0.01–3.13
Consistent with case definition^b^	21	7.40	4.55–11.25	3	1.54	0.30–4.53

COVID-19: coronavirus disease; SARS-CoV-2: severe acute respiratory syndrome coronavirus 2.

^a^ Individuals who reported at least one symptom but whose symptom(s) was not consistent with the Irish case definition for COVID-19 of the time (Table 1).

^b^ Individuals who reported one of the following: fever, cough, shortness of breath, loss sense of smell or taste.

Missing values were regarded as negative for the symptom except for participants for whom no symptom data were available, who were excluded.

### Seroprevalence in healthcare workers

Four of 33 anti-SARS-CoV-2-positive participants self-identified as healthcare workers (HCW) – three in Dublin and one in Sligo – compared with 116/1,700 of seronegative participants. This difference was not statistically significant (p = 0.248). Examining the Dublin and Sligo participant datasets separately, the weighted prevalence of antibodies to SARS-CoV-2 among HCW was almost twice that observed for non-HCW in both sites (in Dublin 5.78% (95% CI: 1.13–16.38) vs 3.00% (95% CI: 1.92–4.45); in Sligo 1.32% (95% CI: 0.03–7.52) vs 0.52% (95% CI: 0.14–1.35)), although it did not reach statistical significance.

## Discussion

This was the first population-based seroprevalence study for SARS-CoV-2 performed in Ireland. It took place 10−14 weeks after the peak of reported COVID-19 cases in Ireland during the first wave, which occurred in the week of 12 April 2020. Therefore, we believe that antibody levels would not have declined by the time the study was conducted in June–July 2020. The seroprevalence estimates for Dublin and Sligo of 3.1% and 0.6%, respectively, were consistent with our expectations of a higher seroprevalence for Dublin than Sligo. Moreover, the ratio of notified laboratory-confirmed COVID-19 cases to numbers of SARS-CoV-2 infections based on the estimated prevalence was relatively consistent between the two counties. The seroprevalence found in these two areas was then used to estimate the national seroprevalence of 1.7%.

Our finding of a seroprevalence of 1.7% for Ireland overall was lower than other national seroprevalence estimates in Europe during that period. Spain, Italy and England, countries that experienced higher levels of infection, reported national seroprevalence estimates of 5%, 2.5% and 6%, respectively [10-12]. Conversely, Hungary, in a similar national cross-sectional serosurvey, reported a lower seroprevalence of 0.68% [9]. Comparing SARS-CoV-2 seroprevalence rates across Europe is challenging, however, due to differences in the methodological approaches taken, including sampling strategies and age. Unlike other studies, our study did not include those aged over 69 years. Merkely et al., the authors of the Hungarian study, noted that restrictive measures to contain the spread of SARS-CoV-2 were implemented early, 12 days after the first case was reported in that country [13]. In Ireland, schools closed 12 days after the first case was notified and a stay-at-home order was announced 16 days later. It is likely that the early introduction of restrictions and widespread compliance contributed to the reduced transmission.

Our finding that the estimated number of SARS-CoV-2 infections in Ireland was only three times higher than the number of notified laboratory-confirmed cases of COVID-19 contrasts with other countries, such as England and the US, where the nationwide seroprevalence estimates suggest that the true number of infections was over 10 times higher than the number detected through PCR testing during the first wave [12,14]. During the week of 20 July 2020, Ireland’s rate for SARS-CoV-2 RNA testing was 1,013 per 100,000 population, putting Ireland seventh in EU/EEA and the UK in terms of the number of tests performed per capita [15]. The two points above suggest that the testing strategies and surveillance systems in place in Ireland are relatively sensitive.

Nine of the 33 participants with antibodies to SARS-CoV-2 did not report symptoms that fit the Irish COVID-19 case definition. Only 3 of 33 seropositive participants reported no symptoms at all. This is much lower than the asymptomatic proportion found in other seroprevalence studies, where around one third of those with antibodies were asymptomatic [10,12]. One explanation for this may be volunteer bias in our study, as those who had experienced symptoms suggestive of COVID-19 since February may have been more likely to participate. In addition, symptoms reported could have been related to other viruses in circulation, such as influenza, leading to an overestimation of those categorised with symptomatic COVID-19.

A key strength of this study was the random selection of individuals who were representative of the population living in Dublin and Sligo, in terms of age and sex. Participants aged less than 70 years living in residential facilities were not excluded, unless advised to cocoon (stay at home as much as possible and limit contact with other people) for a reason other than age, e.g. as a result of a medical condition placing them at high risk from COVID-19.

The participant response rate to the seroprevalence part of our study was 35%, lower than we had anticipated. This study commenced during the period when everyone was encouraged to stay local, work from home where possible, and avoid using public transport. This guidance changed during the course of the study, insofar as more businesses were allowed to re-open, but the use of public transport was still recommended for essential journeys only. Some invitees may have been reluctant to travel for testing due to the ongoing risk of COVID-19 and the restrictions in place, while others may have been unable to attend for other reasons, such as returning to work. Response rates were not equal across the different age groups; younger people (< 40 years), particularly young men, were under-represented. A similar study in Geneva that also collected blood samples for laboratory-based analysis found that 35% accepted the invitation [16], but other studies have reported higher participation rates. The response rate for antibody testing in the Hungarian national serosurvey was 65% [9] and in Spain, 64% of eligible participants provided a blood sample for laboratory-based analysis in the ENE-COVID study [10]. The effect of non-response bias on the seroprevalence estimates in our study is unknown. It could have resulted in an under- or over-estimation of prevalence.

The SCOPI study had some other limitations. Recruitment took place in only two geographic areas, and thus the method to obtain the national estimate could be regarded as relatively crude. Participants under 12 and over 69 years of age were not included. The sampling frame used was not a national register, and based on some comparisons carried out by the Central Statistics Office (CSO), may have over-represented those who are either very young or very old, economically inactive, have a disability or a low level of completed education, when compared with the general population, as measured by the population census in 2016 [7]. While the variation in age profile was addressed by our age–sex stratified sampling approach, the over-representation of other groups may have resulted in an under-representation of individuals who were more likely to be mixing in public, and may have resulted in an underestimate of seroprevalence. There may also have been recall bias given the long interval (4 months) over which participants were required to remember the occurrence of symptoms.

There may be limitations regarding the application of anti-SARS-CoV-2 tests to determine the level of SARS-CoV-2 infection in the population. Historically, serum antibody-based investigations of respiratory infection can be compromised as the infection occurs in the respiratory tract and there is a locally derived mucosal immune response. Therefore, using a serum sample to detect an antibody response is not optimal, especially in those with mild symptoms. It has been observed that anti-SARS-CoV-2 antibodies are detectable more frequently in those patients with more severe systemic infection. It is possible that individuals who had milder disease or were asymptomatic are less likely to develop a detectable antibody response in serum [2]. Therefore, some previous mild infections may not have been detected. Even among those with a clear symptomatic infection, a small percentage do not have serological evidence of SARS-CoV-2 infection [2]. Emerging evidence suggests that individuals vary in their antibody response to SARS-CoV-2, both in the level of antibody produced and to which viral protein target, e.g. nucleoprotein or spike protein, their antibodies are predominantly directed. Therefore, we decided to initially test using an anti-nucleoprotein assay and confirm any sample above or within 25% of the cut-off, with an anti-spike protein assay. We believe the approach taken in this study improved the overall accuracy of the serological results generated. Evidence has emerged after this study was completed to suggest that the sensitivity of the Abbott assay declines with time, by around 30% after more than 81 days following a positive SARS-CoV-2 PCR test [17,18]; however, our study probably occurred soon enough after the peak in the first wave so that the effect was limited.

Our experience of conducting a population-based seroprevalence study during the active stage of a pandemic identified many logistical challenges. Organising recruitment and serological testing of participants during the pandemic was difficult and resource-intensive. These challenges, and the ongoing and evolving nature of the pandemic as evident since the SCOPI study was carried out, underline the need for a sustainable means of obtaining seroprevalence data. This has resulted in the establishment of a national serosurveillance programme in Ireland [19], using a residual sera sampling approach, as is used in other countries such as Scotland [20].

## Conclusions

The estimated low prevalence of anti-SARS-CoV-2 found in the SCOPI study suggests that the vast majority of people living in Ireland were unlikely at that time to have been infected with SARS-CoV-2 and remained susceptible. As SARS-CoV-2 was a recently emerged virus at the time of our study, the duration of detectable antibody post-infection was not known. This highlighted the continued importance of public health measures, including physical distancing, respiratory etiquette, hand hygiene, and the use of face coverings, pending the widespread availability of vaccination, and effective treatments. With increases in cases, hospitalisations and deaths in Europe in October and November 2021, driven by the SARS-CoV-2 Delta variant, these public health measures, in combination with vaccination and booster doses for adults, remain essential in order to control transmission and respond effectively to the pandemic.

## Note

Preliminary results of this study were released by the HSE on 20 August 2020. This report is available at https://www.hpsc.ie/a-z/respiratory/coronavirus/novelcoronavirus/scopi. These results were shared with the WHO in an anonymised format to add to the global knowledge base on the seroprevalence of SARS-CoV-2.
